# Two Strains of *Lymnaea stagnalis* and the Progeny from Their Mating Display Differential Memory-Forming Ability on Associative Learning Tasks

**DOI:** 10.3389/fnbeh.2017.00161

**Published:** 2017-09-11

**Authors:** Hiroshi Sunada, Yuki Totani, Ryota Nakamura, Manabu Sakakibara, Ken Lukowiak, Etsuro Ito

**Affiliations:** ^1^Kagawa School of Pharmaceutical Sciences, Tokushima Bunri University Sanuki, Japan; ^2^Department of Biology, Waseda University Tokyo, Japan; ^3^Research Organization for Nano and Life Innovation, Waseda University Tokyo, Japan; ^4^Hotchkiss Brain Institute, University of Calgary Calgary, AB, Canada; ^5^WASEDA Bioscience Research Institute in Singapore Singapore, Singapore; ^6^Lipid Science and Aging Research Center and Center for Stem Cell Research, Kaohsiung Medical University Kaohsiung, Taiwan

**Keywords:** aerial respiratory operant conditioning, conditioned taste aversion, F_1_ cross, *Lymnaea*, interstrain differences

## Abstract

The pond snail *Lymnaea stagnalis* learns and forms long-term memory (LTM) following both operant conditioning of aerial respiratory behavior and classical conditioning of taste aversive behavior. In the present study, we examined whether there are interstrain differences in the ability to form LTM following these two types of conditioning. A strain of *Lymnaea* (TC1) collected in Alberta, Canada exhibits superior memory-forming ability following aerial respiratory operant conditioning compared to a laboratory-reared strain of *Lymnaea* from Netherlands known as the Dutch strain. We asked whether the offspring of the Canadian TC1 and Dutch snails (i.e., filial 1 (F_1_) cross snails) would have the superior memory ability and found, rather, that their memory ability was average like the Dutch snails. That is, the Canadian TC1 snails have superior ability for LTM formation following aerial respiratory operant conditioning, but the Dutch and the generated F_1_ cross have average ability for memory forming. We next examined the Canadian TC1, Dutch and F_1_ cross snails for their ability to learn and form memory following conditioned taste aversion (CTA). All three populations showed similar associative CTA responses. However, both LTM formation and the ratio of good-to-poor performers in the memory retention test were much better in the Dutch snails than the Canadian TC1 and F_1_ cross snails. The memory abilities of the Canadian TC1 and F_1_ cross snails were average. Our present findings, therefore, suggest that snails of different strains have different memory abilities, and the F_1_ cross snails do not inherit the memory ability from the smart strain. To our knowledge, there have been a limited number of studies examining differences in memory ability among invertebrate strains, with the exception of studies using mutant flies.

## Introduction

The pond snail *Lymnaea stagnalis* is a useful animal model for investigating the causal, neuronal mechanisms of learning and memory from the behavioral to molecular levels (Lukowiak and Syed, 1999; Benjamin et al., 2000; Hatakeyama et al., 2006; Kojima et al., 2015; Sunada et al., 2017a,b). *Lymnaea* can be both operantly (aerial respiratory behavior) and classically (feeding behavior) conditioned (Kemenes and Benjamin, 1989; Kojima et al., 1996; Lukowiak et al., 1996). When snails placed in an aquatic, hypoxic environment received a tactile stimulus to the pneumostome area every time they attempted to breathe, the snails learned not to breathe, and the number of stimuli received in a memory test session was significantly lower than that in the training session (Lukowiak et al., 1996, 2000; Lukowiak and Syed, 1999). The early studies performed by the Lukowiak laboratory utilized an inbred laboratory-reared strain of *Lymnaea* from Netherlands (hereinafter, the Dutch strain; see Van Der Steen et al., 1969) for the aerial respiratory operant conditioning. In the Dutch strain, two 0.5 h training sessions with a 1 h interval between them were required for the snails to form long-term memory (LTM) persisting for at least 24 h (Lukowiak et al., 2000). However, some naturally occurring strains of *Lymnaea* form memory faster and better than the Dutch strain (Orr et al., 2008, 2009; Dalesman and Lukowiak, 2012). In these so-called smart snails (e.g., Canadian TC1 strain; see below for details), LTM is formed after only a single 0.5 h training session (Shymansky et al., 2017).

Having shown that there are differences in learning and memory-forming abilities following aerial respiratory operant conditioning, in a recent study we sought to begin to determine the cost of being smart. Hughes et al. (2017) showed that one apparent cost of possessing the smart phenotype was that the smart snails were less able to cope with stress than average snails. The stressors used in the Hughes et al. (2017) study were thermal, resource restriction combined with food odor, predator detection and tissue injury (shell damage). Their results suggested that a stressor or a combination of stressors act to enhance memory in average snails but obstruct memory formation in smart snails. These results are consistent with the Yerkes-Dodson/Hebb law, resulting that smart snails are more easily stressed than average snails (Yerkes and Dodson, 1908; Hebb, 1955; Ito et al., 2015).

In the present study, we ask whether there are other costs or benefits of possessing the smart phenotype as determined by the studies of operant conditioning of aerial respiration (e.g., Hughes et al., 2016; Shymansky et al., 2017). Previously, attempts have been made to study this question using the fruit fly *Drosophila*. However, almost all these studies utilized different mutants (Tully, 1996). It may be better to examine this question using different strains of a single animal species rather than mutants. This is because we are able to perform behavioral experiments on different known strains and then to find the causes, for example genetic drift. Genetic drift has been suggested to account for significant differences in European *Lymnaea stagnalis* populations (e.g., how they respond to different levels of copper in the water) that occur (Puurtinen et al., 2004a,b, 2007; Côte et al., 2015). Changes in learning and memory forming ability due to genetic drift possibly have a more important biological meaning than those due to mutation. Thus, we used different strains of *Lymnaea* to better answer these questions.

Two established strains of *Lymnaea* were used in the present study: the Dutch strain and the Canadian TC1 strain. As a third population, the filial 1 (F_1_) generation resulting from a cross of the Dutch and Canadian TC1 snails was also used. These three populations were bred in a laboratory (i.e., not freshly collected). The Dutch strain was isolated in the 1950s from freshly collected snails (see Van Der Steen et al., 1969). The Canadian TC1 strain was collected in 2010 (Braun et al., 2012) and has been bred in Alberta, Canada and in Kagawa and Tokyo for many generations. The F_1_ cross population was bred in Tokyo, Japan.

Studies on the classical conditioning of taste aversive behavior (i.e., conditioned taste aversion, CTA) in Dutch snails were previously performed at the Ito laboratory. In CTA experiments, an appetitive stimulus (e.g., sucrose) is used as the conditioned stimulus (CS), and an application of the CS to the lips increases the feeding response (i.e., the number of bites). An aversive stimulus (e.g., electric chock) is used as the unconditioned stimulus (US), and the US causes the snails to immediately cease feeding. In the CTA-training procedure, the CS is paired with the US. After repeated CS-US temporal pairings, the CS no longer elicits feeding, and this CTA persists for at least a month (Kojima et al., 1996; Ito et al., 1999, 2013).

Recent studies at the Ito laboratory have used an automated learning apparatus for CTA (see “Materials and Methods” Section for its details; Takigami et al., 2016). We have obtained good results in Dutch snails using a 5-s presentation of a 100 mM sucrose (the concentration in the reservoir) as the CS and a 0.2-s presentation of a 9 V electric shock as the US. The pond water always flowed at the speed of 200 ml/min, whereas the sucrose solution flowed at 1 ml/s. Thus, the concentration of sucrose in the reservoir is higher than the concentration sensed by the snails because the sucrose solution was diluted with the continuous pond water flow. On the other hand, no attempts have been made to examine the learning ability of CTA in the Canadian TC1 snails.

*Lymnaea stagnalis* is a species distributed across Europe and North America (Mozley, 1939). A mitochondrial ribosomal RNA study suggested that there are genetically distinct populations of *Lymnaea stagnalis*, including in Germany and Italy (Remigio and Blair, 1997). The sequence divergence and genetic distance between these two populations (i.e., German and Italian populations) were greater than those between some separate species of snails. Support for such a conclusion came from a series of studies by a Finnish group studying *Lymnaea stagnalis* in eight different ponds/shallow bays of larger lakes within 20 km of each other in Finland (Puurtinen et al., 2004a,b, 2007). They concluded that there is detectable genetic variability between these populations. This conclusion was also supported in a more recent study concerning the genetic variation in copper tolerance of *Lymnaea stagnalis* (Côte et al., 2015). Random genetic drift was hypothesized to explain the observed genetic divergence. A similar conclusion was drawn in a study of 14 different populations of *Lymnaea stagnalis* collected in Belgium, Netherlands and Germany (Bouétard et al., 2014). Thus, rather than studying differences in learning and memory in mutants (Tully, 1996), we propose to study interstrain differences that are thought to have arisen through normal genetic drift.

In the present study, we both operantly condition and classically condition the above-mentioned two strains and their common offspring (F_1_ cross snails) of *Lymnaea stagnalis*: the Dutch strain, the Canadian TC1 strain and the F_1_ cross snails resulting from their mating. We measure their abilities to learn and form memory, and we examine whether the strain that exhibits better performance in the aerial respiratory operant conditioning task also exhibits better ability to form LTM following the CTA classical conditioning. That is, we examine whether the same strain is “smarter” in terms of both operant and classical conditioning. Further, we established their common offspring (F_1_ cross snails) to examine whether the F_1_ cross snails inherit the memory ability from the smart strain or not.

## Materials and Methods

### Snails

We used two known strains and an F_1_ cross obtained by mating snails from the two strains (F_1_ cross snails) of *Lymnaea stagnalis* (Linnaeus, 1758) in the present study. The first was the Dutch strain, the standard laboratory strain used in studies performed worldwide. This strain was established from snails collected in a polder in Utrecht, Netherlands in the 1950s and continues to be maintained at the Vrije Universiteit Amsterdam in Netherlands (see Van Der Steen et al., 1969). The populations of Dutch snails used in the present study were established in Canada and Japan using snails derived from the Vrije Universiteit Amsterdam colony in 1980s and 1990s, respectively. The second strain used was the Canadian TC1 strain, which was collected from a pond adjacent to the Trans-Canada Highway, Alberta, Canada (at 51.07 N, 114.39 W; see Braun et al., 2012). This Canadian TC1 population was transferred to Japan and maintained separately in dechlorinated tap water as a substitute for pond water under a 12:12 light-dark cycle at around 20°C. Finally, the third was derived from eggs laid by mating the Dutch and Canadian TC1 snails (see below). We termed this population the F_1_ cross. All the snails were fed *ad libitum* on a kind of turnip leaf (*Brassica rapa var. peruviridis*: Komatsuna [in Japanese]) and a spiral shell food (Nisso, Saitama, Japan) every other day (Otsuka et al., 2013). The snails with *a* <5 mm shell were fed with goldfish food originated from vegetables (Kyorin: Hiraki, Himeji, Japan). The snails with a *ca*. 20 mm shell length were used for the behavioral experiments (Yamagishi et al., 2015). The generation time for these two strain snails and their offspring snails is almost 2 years.

### F_1_ Cross Snails

The Dutch and the Canadian TC1 snails were reared in separate aquaria as described above. One immature *Lymnaea* individual with a *ca*. 10 mm shell length was selected from each tank, and these two snails were reared together in a separate aquarium. Because snails do not become sexually mature until obtaining a shell length of 15 mm, these two snails sexually immature (Yamanaka et al., 1999; Sadamoto et al., 2000). Copulation between the snails was observed, and selfing was not observed. Thus, the eggs laid were the result of the mating between the two strains. The hatchlings in these aquaria were designated the F_1_ cross snails. Further, we obtained the F_1_ cross snails from the combination of different Dutch snails and different Canadian TC1 snails. That is, we purposely obtained F_1_ cross snails with great genetic variable. In preliminary experiments, we had already confirmed that F_1_ cross snails can bear eggs and that the fry hatched from these eggs can be normally grown.

### Aerial Respiratory Operant Conditioning

**Figure 1:**
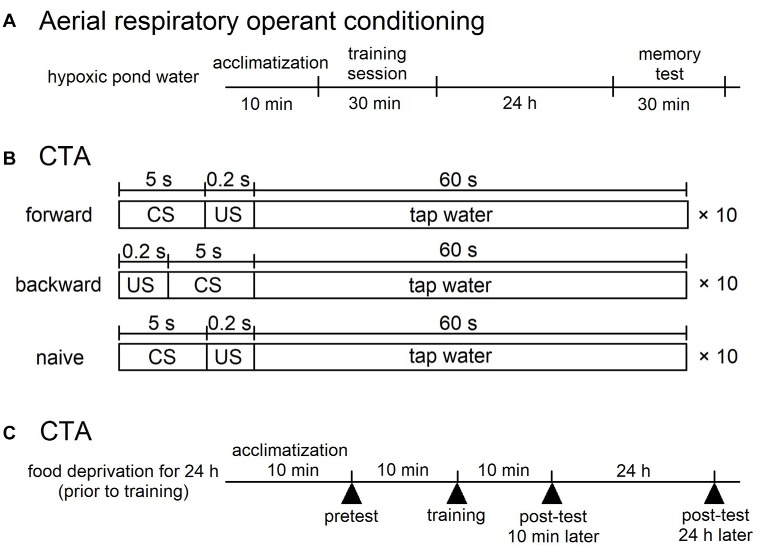
Training procedures for aerial respiratory operant conditining and conditioned taste aversion (CTA) in *Lymnaea*. **(A)** Aerial respiratory operant conditining. The pond water was made hypoxic (≤5% O_2_) by bubbling with N_2_ before training, and bubbling was then continued throughout training. Snails were acclimatized for 10 min before the training session. During the 0.5 h training session, each time a snail attempted to open its pneumostome at the water’s surface, the pneumostome was gently poked. For memory test, the protocol used for 0.5 h training session was repeated at 24 h after the training session. **(B,C)** CTA. **(B)** For forward conditioning (i.e., CTA training), snails were subjected to 10 pairings of sucrose administration (100 mM, conditioned stimulus, CS) for 5 s and electric shock (9 V, 0.4 μA, unconditioned stimulus, US) for 0.2 s. The inter-trial interval was 65.2 s. For backward conditioning, the stimuli were reversed. That is, 10 pairings of application of electric shock (US) for 0.2 s and application of sucrose (CS) for 5 s were used. The inter-trial interval was also 65.2 s in the backward confitioning. For naive control experiments, the CS and the US were replaced with the application of DW. The timng was the same as that of the forward training. **(C)** The time schedule is shown for training. Snails were deprived of food for 1 day before training (except Figure 5). A pretest and two post-tests were performed to count the number of feeding responses to sucrose (bites/min) for 1 min after the application of CS for 5 s. One post-test was performed 10 min after training, and the other post-test was done 24 h after training.

The snails were kept in aerated artificial pond water (a 1% concentration of Instant Ocean; Spectrum Brands, Middleton, WI, USA; with 80 mg/l CaCl_2_) for the aerial respiratory operant conditioning. The training procedure was identical to that described previously (Dalesman and Lukowiak, 2012). To increase the occurrence of aerial respiration, the artificial pond water in a beaker was made hypoxic (≤5% O_2_) by vigorously bubbling with N_2_ for 20 min before training commenced; bubbling was then continued at a reduced rate throughout training to maintain hypoxic conditions (Figure 1A). Snails were replaced into the breaker and allowed to acclimate for 10 min before the training session. This acclimation period was then followed by a 0.5 h training period. During the training period, each time a snail attempted to open its pneumostome (i.e., breathing tube) at the water’s surface, the pneumostome was gently poked using a sharpened wooden stick. This caused the snail to withdraw its pneumostome, but not its whole body to withdraw. To test for LTM, the protocol used for 0.5 h training session was repeated at 24 h after the training session.

### CTA Classical Conditioning

We used a *de novo*, automatic training apparatus that was developed at the Sakakibara laboratory of Tokai University (Takigami et al., 2016), with slight modifications. In this conditioning apparatus, the pond water, which was propelled by a water pump, constantly flowed on snails at a speed of 200 ml per min. We could thus easily supplemented the flowing water with 100 mM sucrose (the CS) and delivered it to the snails for 5 s. The flow of sucrose (1 ml/s) into the test chamber began immediately when the pump was turned on. The amount of sucrose administered to the mouth of an individual snail in this manner was estimated to be 20–30 mM. Fluids were drained from the chamber via an overflow pathway. The US consisted of a 9 V, 0.4 μA electric shock delivered by a dry cell battery and applied for 0.2 s via a 2-channel electronic stimulator (DPS-1200D; Dia Medical System, Tokyo, Japan) or a microcomputer (Arduino Holdings, New York, NY, USA). The US was strong enough to immediately terminate the feeding behavior before the snail withdrew into its shell, but it was not harmful to the snail (Ito et al., 2015). Both the CS and US were delivered to the snail from signals generated from an electronic stimulator. This stimulator can generate any arbitral timing signals with any duration, delay and repetition number. The snail’s feeding behavior was observed by a human experimenter. Each test chamber consisted of a 50 ml culture flask bottle equipped with an in and out flow path.

Snails were first challenged with a pretest by the application of sucrose (CS; Murakami et al., 2013). The number of bites was recorded in the 1-min interval in pond water after the 5-s application of the CS. Twenty-four hours later, the snails were again subjected to the conditioning and control procedures in the same apparatus as used for the pretest. In the CTA training procedure, we paired the CS with the US, and the snails received 10 paired presentations of the CS-US (forward; Figure 1B). Controls included a backward-conditioned (US-CS) group and a naive group to validate associative learning (Figure 1B). For the naive control group, only pond water was applied to the lips instead of the CS and US. In every instance, the inter-trial interval was set as 65.2 s, because snails required this time to recover from the withdrawal response caused by the electrical stimulus (i.e., the US). The conditioning procedure was completed within 1 day. In the post-test sessions, snails were again challenged with the CS, and the number of bites was counted in the 1-min interval in pond water after the 5-s application of the CS. The time schedule of these protocols is shown in Figure 1C.

Based on our previous findings (Sugai et al., 2007), we set a performance cutoff in the post-test sessions to distinguish between good and poor performers. A snail possessing good learning and LTM (i.e., a good performer) will not open its mouth (i.e., a bite) following presentation of the CS. However, some snails bite by chance (i.e., spontaneously) in the absence of any delivered stimulus (Kojima et al., 1996). Such spontaneous openings occur at a rate of about one per minute. Thus, we defined a good performer as a snail that made 0–1 bites/min during the post-test session in response to presentation of the CS. Poor performers were thus defined as snails that made ≥2 bites/min in response to the CS during the post-test session. In other words, if a snail completely learns and forms memory of CTA, it does not bite ≥2 times/min in response to the sucrose CS. We thus set the threshold at 1 bites/min to distinguish a good performer from a poor performer. The behavioral experiments were performed in the morning, because it has been shown that snails exhibit better learning ability in the morning than at other times (Wagatsuma et al., 2004).

For both types of conditioning (i.e., aerial respiratory operant conditioning and CTA classical conditioning), the training procedures used would allow us to easily distinguish between snails exhibiting enhanced cognitive ability (Shymansky et al., 2017). Those that did not exhibit such enhancement were termed average snails (Hughes et al., 2017).

### Data Analysis

The data are expressed as the mean ± SEM. Significant differences were examined at *P* < 0.05. A paired *t*-test was performed between the number of attempted openings in the training session and that in the memory test in the operant conditioning of aerial respiration. For there to be memory present, the number of attempted pneumostome openings in the memory test session had to be significantly less (i.e., *P* < 0.05) than the number of attempted openings in the single 0.5 h training session. Following the CTA training procedure, a one-way ANOVA followed by a *post hoc* Scheffé’s test or a two-way repeated measures ANOVA having one between subjects factor (strain: Dutch snails, Canadian TC1 snails and F_1_ cross snails) and one within-subjects factor (time course: pretest, 10 min post-test and 24 h post-test) followed by a *post hoc* Holm’s test was used to determine whether memory was present (i.e., whether there was a significant difference in the number of bites compared to the pretest response; *P* < 0.05). When the variances of within-subjects factor were not equal, the degrees of freedom were adjusted by Greenhouse-Geisser’s epsilon. The ratio of good to poor performers was estimated by a *χ*^2^ test to detect significant differences at *P* < 0.05. The computer software used was R (version 3.3.1).

## Results

### Aerial Respiratory Operant Conditioning

**Figure 2:**
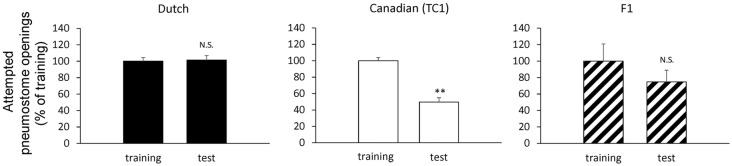
Comparison of the pneumostome opening attempts between the training session (training) and the memory test (test) in the Dutch snails (black, *N* = 60), the Canadian TC1 snails (white, *N* = 60) and the F_1_ cross snails (hatched, *N* = 28) in the operant conditioning of aerial respiratory behavior. The number of pneumostome opening attempts was counted over 0.5 h during the training session and the memory test 24 h later. The data are shown in percentages, indicating that the results of F_1_ cross snails resembled those of the Dutch snails. We calculated the statistical significant using the raw data. **P < 0.01; N.S., not-significant. The statistical analysis was performed usign a paired *t*-test.

First, we performed experiments to show the difference between the smart Canadian TC1 snails and the average Dutch snails in forming memory following operant conditioning of aerial respiratory behavior. In each cohort of snails (*N* = 60 each), the snails were subjected to a 0.5 h training session, then an interval of 24 h and a 0.5 h memory test. The Dutch strain was unable to form LTM following the single training session (i.e., the number of attempted openings in the memory test was not significantly different than that in the training session), whereas the Canadian TC1 strain exhibited LTM (i.e., there was a significant difference in the number of attempted openings in the memory test compared to the training session; *P* < 0.01). To easily compare the data, we plotted the ratios of a change as a percentage of the initial responses in the training session. These data are presented in Table 1 and Figure 2 and are consistent with the previous published data (e.g., Braun et al., 2012).

**Table 1:** Comparison of learning and memory-forming abilities in two strain snails and their offsprings in *Lymnaea stagnails*.

Associative learnings	Dutch snails	Canadian (TC1) snails	F_1_ cross snails
Aerial respiratory operant conditioning	Average	Smart (*P* < 0.01)	Average
CTA at 10 min post-test			
(1) memory formation	Smart	Smart	Smart
(2) ratio of good and poor performers	Not signifaicant	Not signifaicant	Not signifaicant
CTA at 24 h post-test			
(1) memory formation	Smart (*P* < 0.05)	Average	Average
(2) ratio of good and poor performers	High (*P* < 0.01)	Low	Low

We next examined the behavioral phenotype of the F_1_ cross snails following aerial respiratory operant conditioning. We found that the F_1_ cross snails did not show LTM formation following the single 0.5 h training session (*P* > 0.05; Figure 2). That is, the F_1_ cross snails showed an average ability for aerial respiratory operant conditioning. However, as can be seen, the number of attempted openings in the training session of the F_1_ cross cohort of snails (*N* = 28) was much smaller than the number of attempted openings in either the Dutch or the Canadian TC1 cohorts. These data show that whereas the Canadian TC1 snails exhibited the smart phenotype, neither the Dutch nor the F_1_ cross snails met the criteria to be considered smart.

### CTA Classical Conditioning

**Figure 3:**
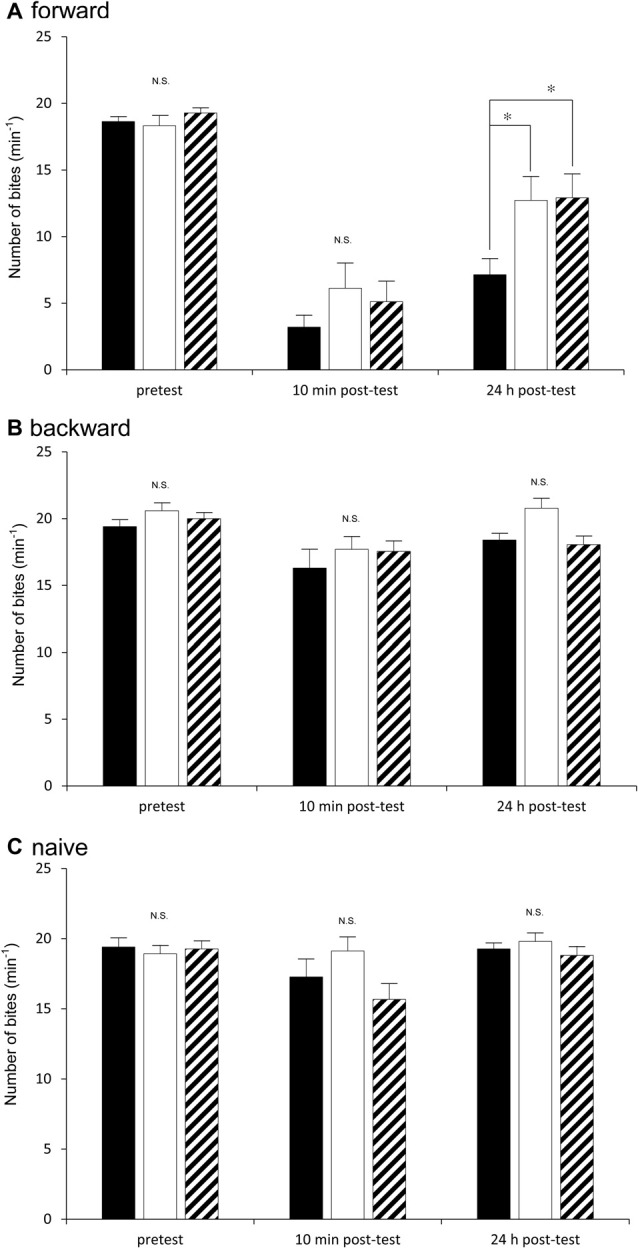
Comparison of the feeding responses to the sucrose CS among the Dutch snails (black), the Canadian TC1 snails (white) and the F_1_ cross snails (hatched) in the classical conditioning trial of taste aversion behavior. The feeding response was examined at pretest, 10 min post-test and 24 h post-test in response to the CS. **(A)** Forward conditioning (i.e., CTA training) for the three cohorts (i.e., the Dutch, the Canadian TC1 and the F_1_ cross snails) was performed. The results showed that (1) there was a significant difference in the feeding responses shown in **(A)** (two-way repeated measures ANOVA: for strains *F*_(2,91)_ = 3.84, *P* < 0.05; for time course *F*_(2,182)_ = 118.18, *P* < 0.01; for interaction *F*_(4,182)_ = 2.66, *P* < 0.05). The simple effect test showed a significangt difference for strains at 24 h post-test (*F*_(2,91)_ = 5.04, *P* < 0.01). Further, a Holm’s multiple comparison showed *P < 0.05 between the Dutch and F_1_ cross snails; *P < 0.05 between the Dutch and Canadian TC1; and *P* > 0.05 between the Canadian TC1 and F_1_ cross snails. (2) There were no significant differences in the feeding response to the sucrose CS at the pretest (*F*_(2,91)_ = 6.07, *P* > 0.05). (3) The feeding responses to the CS were significantly suppressed in all three cohorts (for example, *P* < 0.01 for the Canadian TC1 snails), but there were no significant differences among the three cohorts at the 10 min post-test (*F*_(2,91)_ = 1.27, *P* > 0.05). **(B)** Backward conditioning (pairings of the US first and then the CS) for the Dutch, the Canadian TC1 and the F_1_ cross snails showed that (1) there were no significant differences among the three cohorts at the pretest, the 10 min post-test and the 24 h post-test (two-way repeated measures ANOVA: for strains *F*_(2,52)_ = 0.42, *P* > 0.05); but (2) there were significant differeces in the feeding responses shown in B (two-way repeated measures ANOVA: for time course *F*_(2,79.32)_ = 9.22, *P* < 0.01. Further, a Holm’s multiple comparison showed *P < 0.05 between the pretest and 10 min post-test; *P < 0.05 between the pretest and 10 min post-test; *P < 0.05 between the 10 min post-test and 24 h post-test). There were no significant interaction (two-way repeated measures ANOVA: for interaction *F*_(3.05,79.32)_ = 0.81, *P* > 0.05). **(C)** The naive snails also showed that (1) there were no significant differences among the three cohorts at the pretest, the 10 min post-test and the 24 h post-test (two-way repeated measures ANOVA: for strain *F*_(2,52)_ = 0.81, *P* > 0.05). (2) There were no significant differences in the feeding responses (two-way repeated measures ANOVA: for time course *F*_(1.57,81.69)_ = 2.95, *P* > 0.05). There were no significant interaction (two-way repeated measures ANOVA: for interaction *F*_(3.05,79.32)_ = 0.81, *P* > 0.05).

We next examined how the three cohorts of snails (i.e., the Dutch, the Canadian TC1 and the F_1_ cross snails) performed in a CTA trial. Previous reports demonstrated that the best learning and memory scores were obtained when the snails were food-deprived for 1 day before training (Sugai et al., 2007; Mita et al., 2014). Thus, in the first experiment using the CTA training procedure, we food-deprived all the snails for 1 day. In the pretest, the number of bites was recorded after application of the sucrose CS. There were no significant differences (*P* > 0.05) in the feeding responses elicited by the CS among the three cohorts of snails (i.e., the Dutch, the Canadian TC1 and the F_1_ cross snails) in the pretest session (Figure 3A). That is, each of the cohorts responded to the sucrose stimulus in a similar fashion. We then tested the three cohorts 10 min after CTA training (i.e., the 10 min post-test). The CS-elicited feeding responses in all three cohorts were significantly decreased in comparison with those of the pretest (*P* < 0.01). In addition, there were no significant differences in the number of feeding responses elicited by the CS among the three cohorts (*P* > 0.05, Figure 3A). That is, all three cohorts showed similar associative learning following CTA conditioning.

When we tested for LTM 24 h after CTA training (i.e., the 24 h post-test), we found that memory was present in all three cohorts. That is, in all three cohorts the number of bites elicited in the 24 h post-test was significantly smaller than that in the pretest (*P* < 0.01, Figure 3A). Thus, all the cohorts were capable of forming CTA-LTM following the conditioning procedure. However, we found that the feeding response in the Dutch snails in the 24 h post-test was significantly smaller than those of the Canadian TC1 and the F_1_ cross snails (*P* < 0.05, Figure 3A). That is, the Dutch snails formed better CTA-LTM than the other two cohorts of snails. The results indicated that the Dutch snails exhibited the smart phenotype, whereas the Canadian TC1 and the F_1_ cross snails showed average intelligence in the CTA classical conditioning trial.

In the control behavioral experiments (i.e., the backward conditioning and the naive control procedures), there were no significant differences in the feeding responses to the sucrose CS among the three cohorts at either the pretest, the 10 min post-test or the 24 h post-test (Figures 3B,C). These results support the validity of the CTA training shown in Figure 3A and highlight the different learning abilities of the Dutch and the Canadian TC1 snails at the 24 h post-test after the CTA training procedure.

### Good and Poor Performers in the CTA Trial

**Figure 4:**
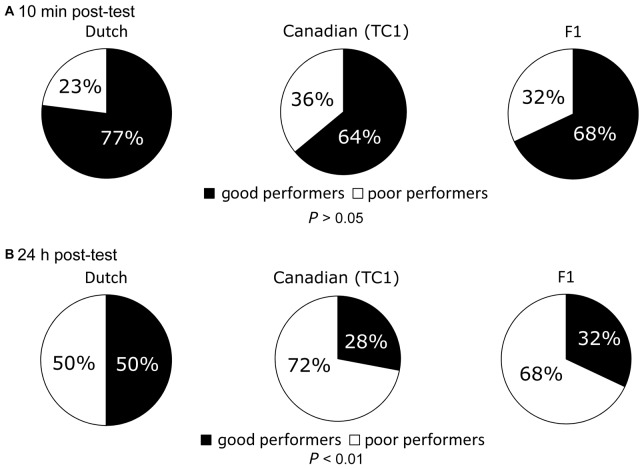
Comparison of the ratio between the good and poor performers at the memory retention tests among the Dutch, the Canadian TC1 and the F_1_ cross snails. Pie charts express the ratio (percentage) of the good and poor performers. **(A)** At 10 min post-test, no significant differences were found (*P* > 0.05) in the ratios of good performers (black) or poor performers (white) among the three cohorts. **(B)** At 24 h post-test, there was a significant difference among the three cohorts (*P* < 0.01). That is, the Canadian TC1 and the F_1_ cross snails exhibited poor formation of CTA-long-term memory (LTM).

To further confirm that the Dutch snails had better LTM than the other two cohorts, we examined the ratio between the good and poor performers in response to the CS after both the 10 min and 24 h post-test sessions in the Dutch, Canadian TC1 and F_1_ cross snails (Figure 4). As described in the “Materials and Methods” Section, we defined a good performer as a snail that made 0–1 bites/min during the post-test session in response to the CS. A poor performer was thus defined as a snail that made ≥2 bites/min in response to the CS during the post-test session. At the 10 min post-test after the CTA training, there were no significant differences in the learning performance among the three cohorts (Figure 4A, *P* > 0.05). Thus, each of the three cohorts performed equally well on the 10 min post-test. This result was in agreement with the results of the feeding responses (Figure 3A).

We also compared the ratio between the good and poor performers in response to the sucrose CS at the 24 h post-test in the three cohorts (Figure 4B). The ratio of good and poor performers in the Canadian TC1 and the F_1_ cross snails was significantly lower than that of the Dutch snails (*P* < 0.01, Figure 4B). Again, these data showed that the Dutch snails were more successful at forming memory than either of the other two cohorts.

### Food Deprivation as a Stressor Affecting CTA

**Figure 5:**
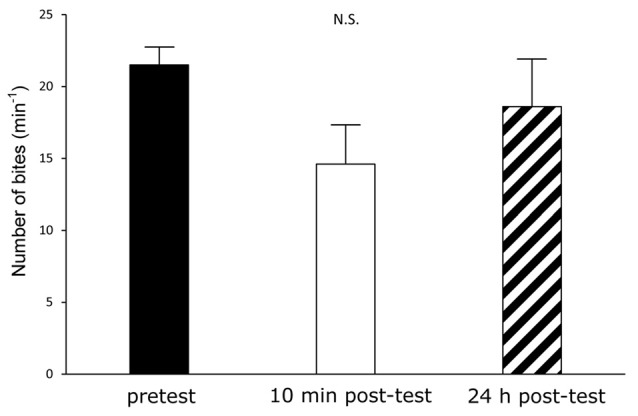
CTA in the Canadian TC1 snails without 24-h food deprivation. If the food deprivation before CTA training had been stressful to the Canadian TC1 snails, we expected that the memory scores would have declined. We therefore trained the food-satiated snails by the CTA training paradigm. However, there was no significant difference among the pretest (black), the 10 min post-test (white) and the 24 h post-test (hatched). That is, the learning and memory were worse in snails in the satiated state than in snails with modest food deprivation (i.e., food deprivation of 1 day), as previously reported.

Finally, we examined the possibility that 1-day food deprivation was a greater stressor for the Canadian TC1 snails (Hughes et al., 2017). According to the Yerkes-Dodson/Hebb inverted-U law, a strong stressor impairs learning and memory, and thus there is an optimal level of arousal for learning and memory (Yerkes and Dodson, 1908; Hebb, 1955; Ito et al., 2015). However, our results using satiated Canadian TC1 snails showed that the feeding behavior was not suppressed at the post-tests after the CTA training (Figure 5). As demonstrated previously in Dutch snails (Aonuma et al., 2016, 2017), the scores obtained in a satiated state are not as good as those obtained in a modestly hungry state (i.e., after 1 day of food deprivation). Thus, 1 day of food deprivation was not a strong stressor for either the Canadian TC1 snails or the Dutch snails.

## Discussion

We here showed that there were significant differences among three distinct populations of *Lymnaea stagnalis* (i.e., the Dutch, the Canadian TC1, and the F_1_ cross snails) with respect to learning and memory-formation abilities following either an operant or a classical conditioning procedure. The Dutch and Canadian TC1 strains exhibited both strengths and weaknesses in their respective ability to form memory with the two different training procedures. The Canadian TC1 snails possessed better memory-forming ability in the aerial respiratory operant conditioning trial than the Dutch and the F_1_ cross snails, whose learning phenotype was average. On the other hand, all three cohorts exhibited associative learning following CTA conditioning. However, the LTM formation in the Dutch snails was much better than that exhibited by the Canadian snails and the F_1_ cross snails. Thus, whereas the Canadian TC1 snails had a superior memory-forming capability following the operant conditioning procedure, this superiority did not translate into forming the best memory on the CTA task. Similarly, the Dutch snails performed better on the CTA task but did not exhibit superior LTM following the operant conditioning procedure. Finally, snails of the F_1_ cross population inherited neither the smart phenotype for operant conditioning from the Canadian TC1 snails, nor the smart phenotype for classical conditioning from the Dutch snails.

To better understand the present findings, we should consider two points: genetic differences between the Dutch and the Canadian TC1 strains; and genetic drift caused by these differences. The genetic differences were the basis for the different memory phenotypes shown above. With respect to the genotype of this species, Mozley (1939) described three sub-species of *Lymnaea stagnalis* in North America based on shell morphology (*Lymnaea stagnalis sanctaemariae*, *Lymnaea stagnalis lillianae* and *Lymnaea stagnalis wasatchensis*). He believed that these three sub-species appeared to be “specially adapted to the conditions under which they lived”. In more recent studies using newer techniques, Remigio concluded that there are genetically distinct populations within *Lymnaea stagnalis* (Remigio and Blair, 1997; Remigio, 2002). This conclusion was also supported by data showing a genetic variation in the tolerance of *Lymnaea* to copper (Côte et al., 2015). In that study, five naturally occurring freshly collected strains from specific ponds as well as three laboratory inbred strains were used. One of those inbreed strains was the Dutch strain that we used here. Further support for the genetically distinct comes from the work performed in Finland on *Lymnaea stagnalis* collected from a number of small lakes, ponds and the bays of larger lakes (Puurtinen et al., 2004a,b, 2007). The authors found detectable genetic variability between those populations of *Lymnaea stagnalis*. Genetic drift also occurred in *Lymnaea stagnalis* populations in outdoor closed mesocosms (Coutellec and Caquet, 2011).

We are uncertain why the F_1_ cross snails performed more poorly than the Canadian TC1 and the Dutch strains in both the aerial respiratory operant conditioning and the CTA classical conditioning trials. There might be genetic variable in F_1_ cross snails, because we obtained the F_1_ cross snails from the combination of different Dutch snails and different Canadian TC1 snails. That is, we purposely obtained the F_1_ cross snails with great genetic variable. Nevertheless, the F_1_ cross snails showed the average ability for learning. In the case of the CTA, one possibility is that there are different levels of dopamine or octopamine in the central nervous system (CNS) of the three populations. We posit this idea because we have previously shown that the memory performance in the Dutch strain following CTA training was negatively correlated with dopamine/octopamine levels in the CNS (Aonuma et al., 2016, 2017). It may therefore be that the F_1_ cross snails have higher levels of CNS dopamine/octopamine compared to the Dutch strains. Further experiments will be necessary to confirm this hypothesis and possibly to elucidate other reasons for the poor performance of this population.

Understanding why different strains of the same species, or why different individuals of the same strain, exhibit different cognitive properties is one of the most important questions to be answered in the field of neuroscience. Such differences have long been noted with inbred mouse strains (Wahlsten et al., 2003; Tipps et al., 2014). In mice, the usual procedure for establishing inbred strains is mating of brother-sister pairs for a minimum of 20 generations, resulting in lines that are roughly 99% genetically identical (Green, 1981). Many attempts are going on to find out some differences in genetics among inbred mouse strains (Graybeal et al., 2014). However, because of the complexity of the mammalian brain compared to the snail CNS and the more complicated behaviors being studied in rodents compared to the simpler behavioral studies in snails, it may be that the answers to these questions will initially be made in the snail. The interstrain difference is rare in learning abilities in invertebrates as shown in the present study, with the exception of the studies using a parasitoid wasp (Froissart et al., 2017) and mutants in *Drosophila* (Tully, 1996).

As noted above, genetic drift has been shown to occur in laboratory-inbreed strains of *Lymnaea stagnalis* (Côte et al., 2015). It is possible that over time the laboratory-bred Canadian TC1 snails will become behaviorally more like the Dutch snails in the aerial respiratory operant conditioning as well as in CTA. We will, however, be able to monitor this both behaviorally and at the single neuron level, as Braun et al. (2012) have previously demonstrated the differences in the activity of a single neuron, RPeD1, between smart and average freshly collected snails. Thus, over time a smart phenotype snail may disappear in laboratory-bred populations for the aerial respiratory operant conditioning.

We consider the results shown for the backward conditioning snails and the naive snails for CTA training (Figures 3B,C). Both the backward conditioning and the naive snails showed that there were significant differences in the feeding responses for time course (*P* < 0.01). That is, siginificant differences were observed between the pretest, the 10 min post-test and the 24 h post-test. This decrease in the feeding response was considered to be due not to the learning effect but rather to the fatigue, because both the backward conditioning snails and the naive snails showed a tendency toward a slight decrease in the feeding response.

The studies using parasitoid wasps by (Hoedjes et al., 2011) showed variation in learning rate and memory dynamics, and described “learning has several ecological costs”. In our present study, however, we have used the same rearing systems and performed the same experiments under the same environmental conditions for the different strains of *Lymnaea*. We should also note that the elapsed time for rearing in our laboratories was so long (at least 7 years). That is, we should consider genetic shifts rather than ecological factors to well understand our present results. Further, the innate preference in different strains of *Lymnaea* for both “air” for the aerial respiratory operant conditioning and “sucrose” for the CTA classical conditioning was not observed. Thus, we do not have the ability to determine if there are ecological costs to the different strains.

In conclusion, the different strains of *Lymnaea stagnalis* have different learning and memory-forming capabilities. Strains that exhibit intelligence on one test will not necessarily do so on another test. The F_1_ cross snails may inherit a worse ability for both aerial respiratory operant conditioning and CTA classical conditioning. The difference in learning and memory-forming ability among the strains of this species may henceforth attract the attention of researchers in learning and memory.

## Author Contributions

HS conducted the CTA experiment and prepared the figures. YT conducted the aerial respiratory operant conditioning and the CTA experiments, analyzed the data and prepared the figures. RN conducted the CTA experiment. MS planned, designed the experiments and completed the manuscript. KL planned, designed the experiments, conducted the aerial respiratory operant conditioning and completed the manuscript. EI planned, designed the experiments, conducted the aerial respiratory operant conditioning, analyzed the data and held the main responsibility for the final manuscript.

## Conflict of Interest Statement

The authors declare that the research was conducted in the absence of any commercial or financial relationships that could be construed as a potential conflict of interest.
